# Influence of Demographic and Reproductive Factors on Cervical Pre-Cancer and Cancer in Bangladesh

**DOI:** 10.31557/APJCP.2020.21.7.1883

**Published:** 2020-07

**Authors:** Ashrafun Nessa, Rowson Ara, Parveen Fatema, Begum Nasrin, Afroza Chowdhury, Kamrul Hasan Khan, Ashim Ranjan Barua, Mohammad Harun Ur Rashid

**Affiliations:** 1 *Department of Gynaeclogical Oncology, Bangabandhu Sheikh Mujib Medical University (BSMMU), Shahbag, Dhaka-1000, Bangladesh. *; 2 *Department of Obstetrics and Gynaecology, BSMMU, Shahbag, Dhaka-1000, Bangladesh. *; 3 *Department of Histopathology, BSMMU, Shahbag, Dhaka-1000, Bangladesh. *; 4 *Institute of Epidemiology, Diseese Control and Research (IEDCR), Directorate General of Health Services, Mohakhali, Dhaka-1212, Bangladesh. *

**Keywords:** Influence, demographic, reproductive, factors, cervical, pre-cancer, cancer, Bangladesh

## Abstract

**Background:**

In Bangladesh, cervical cancer (CC) is the 2nd most common cancer with estimated 8068 new cases and 5,214 deaths every year. It is also revealed that different socio-demographic factors have association with CC. This study was performed to evaluate the colposcopy outcomes and the association of different demographic and reproductive risk factors with cervical pre-cancer and cancer.

**Methods:**

This retrospective cross-sectional study was carried out at the colposcopy clinic of Bangabandhu Sheikh Mujib Medical University (BSMMU) between January 2010 and December 2016.

**Results:**

A total 16147 women attended the colposcopy clinic of BSMMU with VIA positive reports. Among them, 65.73% women were referred from different VIA centers of Dhaka district. Mean age of marriage of the subjects was 16. 93 (**±** 1) and mean age of 1st delivery was 18.45 years (**± **4.10). Almost three-fourth of them were married before 18 years and had their 1st delivery by 20 years. Colposcopy examination of the VIA positive women revealed that 36.7% had CINI, 10.6% had CINII/ III and 7.1% had carcinoma of cervix. Considering CIN as disease the Sensitivity, Specificity, PPV and NPV of colposcopy were found 99.7%, 75.3%, 70.3% and 99.8% respectively. On other hand considering CIN2+ as disease the Sensitivity, Specificity, PPV and NPV of colposcopy were found 73.8%, 92.7%, 64.4% and 95.2% respectively. Statistical analysis revealed that higher age (p=0.000), lower level of education (p=0.007), lower socioeconomic status (p=0.014), higher parity (p=0.001) had individual influence on cervical pre-cancer and cancer.

**Conclusions:**

This study indicated higher age, low level of education, lower socio-economic condition and higher parity as most important socio-demographic factors for developing cervical pre-cancer and cancer in Bangladesh.

## Introduction

Cervical cancer (CC) is the fourth most frequent cancer in women with an estimated 570,000 new cases in 2018 representing 6.6% of all female cancers. Approximately 90% of deaths CC occurred in low- and middle-income countries (Bray et al., 2018). In Bangladesh, CC is the 2nd most common cancer among women, with age-standardized rates (ASRs) for incidence less than the global average (10.6 vs. 13.1/100,000 women) and mortality higher than the global average statistics (7.1 vs. 6.9/100,000 women). It is estimated that every year 8068 new cases of CC are detected in Bangladesh and 5,214 women die of the disease (Ferlay et al., 2018). International Agency for Research on Cancer. Available CC is caused by human papilloma virus (HPV), a sexually transmitted virus, which is one of the most common viral infections of the reproductive tract. Survival of CC patients are strongly determined by stage at diagnosis. Due to the late stage at diagnosis and inadequate management facilities, mortality rates from cancer cervix are high in Bangladesh. 

The Government of Bangladesh (GOB) has developed CC screening program up to district and selected sub-district (upazila) level and Visual Inspection of Cervix with Acetic Acid (VIA) is considered as a method of screening of CC. VIA is performed at Upazila Health Complexes (UHCs), Maternal and Child Welfare Centers (MCWCs), District Hospitals (DHs), Medical College Hospitals (MCHs) and BSMMU by trained Family Welfare Visitors (FWVs), Senior Staff Nurses (SSNs) and Doctors. The programme offers VIA method for ever married women of 30 years and above. Till now about 375 VIA centers has already been established at primary, secondary and tertiary level health care facilities of 64 districts of Bangladesh. Women with VIA-ve report are advised to have repeat test after 3 years and women with VIA+ve reports are being referred to the colposcopy clinic of BSMMU and other government MCHs for evaluation and management (Nessa et al., 2013, Holme et al., 2017, Nessa et al., 2010). The referral centers have facilities for colposcopy, histopathology and management of precancerous condition of the cervix. Almost all cases of CC are caused by certain strains of HPV and have been etiologically linked to both pre-invasive lesions and invasive cervical carcinoma (Lizano et al., 2009). It is also revealed that different socio-demographic and reproductive factors have association with CC. Epidemiological studies conducted during the past 30 years have consistently indicated that CC risk is strongly influenced by measures of sexual activity, number of sexual partners, age at first sexual intercourse and sexual behavior of the women’s male partners (Remschmidt et al., 2013). There were inadequate information for finding out the influence of demographic and reproductive factors on CC risk in Bangladesh. This study was performed to evaluate the influence of different demographic and reproductive risk factors for developing cervical pre-cancer and cancer among women attending the colposcopy clinic of BSMMU with VIA positive reports.

## Materials and Methods

This retrospective cross-sectional and hospital based study was carried out at the colposcopy clinic of BSMMU between January 2010 and December 2016. In the mentioned period, 16,147 women with VIA positive reports attended colposcopy clinic of BSMMU. Data were collected during first and follow-up visits and recorded in the colposcopy registrars at colposcopy clinic by face to face interview from these women. For demographic variables, the districts (residing district of women), age, education of women, occupation of women, occupation of husband and socio-economical status (based on monthly family income) were considered. On the other hand, parity, age of marriage and age of delivery were considered as reproductive variables in this study.

All women had colposcopic examination by experienced colposcopist using standard colposcopes (Karl Kaps Som 52 or Leisegang 1DF) after receiving verbal consent. The results of the biopsies were also recorded in the colposcopy registry books. From January 2012, colposcopy clinic of BSMMU is following Swede score for management of women. Previously modified Reid score used to guide colposcopy diagnosis.

The quality of the colposcopy centres were well monitored, Postgraduate gynaecologists/SSNs from different MCHs/ BSMMU/ DHs received basic colposcopy training for 12 days from BSMMU. During the training “Colposcopy and Treatment of Cervical Intraepithelial Neoplasia: A beginners Manual” on colposcopy and treatment of CIN published by International Agency for Research on Cancer (IARC) was used as the training guideline. Advanced colposcopy training was given to practicing colposcopists. Colposcopists attended a course on ‘Early Detection and Prevention of CC’ at Barshi jointly organized and facilitated by IARC, a Tata Memorial Centre Rural Cancer Project at Nargis Dutt Memorial Cancer Hospital, Barshi, and GOB in 2016. Histological Diagnosis was made on histology findings of specimens collected by punch biopsy forceps/ LEEP in colposcopy suspected CIN cases. All Histology examinations were done in the Department of Pathology of BSMMU and each slide was reviewed by at least two competent Histopathologist. Histology report was done by a board of the department in the cases of diagnostic disparity.

The final diagnosis was done by the histopathology report. Women with cervical intraepithelial neoplasia (CIN) I lesions were advised for follow-up after one year. However, the women were offered treatment if she desired or there was risk of failure of follow up. Women with CIN II or higher lesions were offered treatment during the same visit by loop electrical excision procedure (LEEP) or thermo-coagulation after collection of tissue for histological examination. Women with invasive cancers were referred to gynae-oncology for further management. 

Data were collected retrospectively from the colposcopy registry books and entered into SPSS for analysis. The baseline characteristics of the women were summarized using means and frequencies. Detection rates of CIN I, CIN II, CIN III, Ca-cervix, normal findings, as well as demographic data were calculated. CIN II and III considered as pre-cancer. Sensitivity, specificity, positive predictive value (PPV) and negative predictive value (NPV) were calculated for colposcopy findings considering histopathology findings as gold standard. Low grade squamous intraepithelial lesion (LSIL, also known as CIN1) is now recognized as a histological diagnosis of benign viral replication that should be managed conservatively (Tainio et al., 2018). Under current clinical guidelines recommended by WHO, women with cervical intraepithelial neoplasia (CIN1) are to be monitored (WHO, 2006). The sensitivity, specificity, PPV and NPV were calculated in two conditions; first one was CIN as disease and second one was CIN2+ as disease, in both conditions women with unavailable histopathology results were excluded. The study was approved by the Institutional Review Board of BSMMU.

## Results

Among 16,147 women with VIA positive cards at the colposcopy clinic of BSMMU from January 2010 to December 2016, more than half attended from different VIA and CBE centers of Dhaka (65.7%). Remaining women attended from other nearer districts like Narsingdi (4.7%), Narayanganj (3.5%), B. Baria (3.4%), Gazipur (3.0 %), Manikganj (2.6%), Comilla (2%) and other 57 districts (14.7%). Among the VIA+ve women at BSMMU colposcopy clinic, 9,733 (60.3%) women were referred from VIA and CBE center of BSMMU, 2,832 (17.5%) from different sub-districts (UHCs) and 1,620 (10.0%) from different District Hospitals (Table 1).

Majority of the VIA+ve women 10,927 (67.7%) were between 30-49 years and 1,455 (9.0%) were more than 50 years of age. Though a good number (66.2%) had at least primary (5 years of schooling) education, only 22.6% had secondary (10 years of schooling) education and above. Majority of the women were housewives. Mean age of marriage was 16. 9 years (**±** 3.1) and mean age of 1st delivery was 18.45 years (**±**4.1). About three-fourth of the women were married before 18 years of age (74.4%) and had their 1^st^ delivery by 20 years (76%). The Mean parity of the women was 2.6 (+1.4) (Table 2). 

Among the 16,147 VIA positive women, 7,226 (44.7%) had normal finding during colposcopy examination and cervical biopsy was not collected from this group. A large number of them (36.7%) had low grade lesions (CIN I), 1,712 (10.6%) women had high grade lesions (9.5% CIN II and 1.1% CIN III) and 1,145 (7.1%) had CC (Table 3). 

Calculation of sensitivity, specificity, positive predictive value (PPV) and negative predictive value (NPV) were done considering histopathology findings as gold standard. Considering CIN as disease the Sensitivity, Specificity, PPV and NPV of colposcopy were found 99.7%, 75.3%, 70.3% and 99.8% respectively. On other hand considering CIN2+ as disease the Sensitivity, Specificity, PPV and NPV of colposcopy were found 73.8%, 92.7%, 64.4% and 95.2% respectively (Tables 4 and 5).

**Figure 1:**
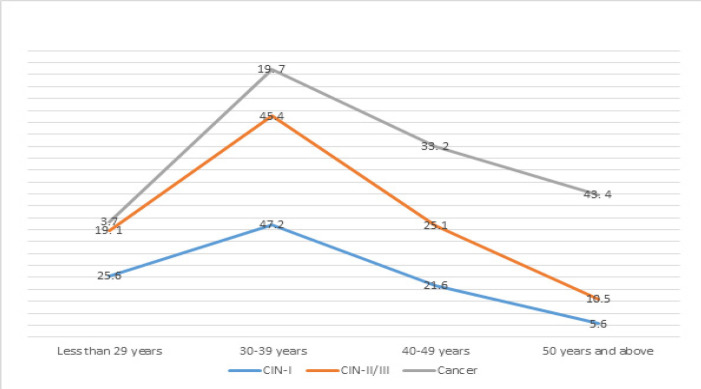
Cervical Pre-Cancer and Cancer among Different Age Groups. The occurrence of cervical pre-cancer and cancer among the women of different age group were shown in Figure 1. We found that the highest 45.4% pre-cancer among the women of 30 -39 years. On the other hand, there were highest 43.4% occurrence of cancer after 50 years.At or after 30 years, the rates of cervical pre-cancer declined whereas cervical cancer incidence became high among the women of 50 years and above

In the age group of less than 29 years, 19.01% women developed high grade pre-cancers, this increased to 45.42% in the 30 -39 years, declined to 25.06% during 40-49 years and further declined to 10.51% after 50 years of age. On the other hand, there was continuous rising trend in case of CC, starting from 3.67% in the age group of less than 29 years, to 43.44% after 50 years of age. The occurrences of cervical pre-cancer and cancer were more among the women of 30-49 years. After the age of 35, the proportion of both low grade and high grade lesions declined rapidly whereas CC remained high among the women of 50 years and above (Figure 1).

The independent influence of different demographic and reproductive characteristics of the women on development of cervical pre-cancer and cancer was assessed (Table 6). Logistic regression analysis revealed that higher age (p=0.000), lower level of education (p=0.007), lower socioeconomic condition (p=0.014), higher parity (p=0.001) had individual influence on development of cervical pre-cancer and cancer. On the other hand marriage and 1st childbirth at younger age did not show independent influence on developing CIN or CC.

Highly significant association were found between education and CIN (X^2^= 63.694; df=1; p<0.001), age of marriage and CIN (X^2^= 24.023; df=1; p<0.001) and parity and CIN (X^2^= 20.338; df=1; p<0.001). Insignificant association were found between age and CIN (X^2^= 0.984; df=1; p=0.321), socio-economic status and CIN (X^2^= 2.421; df=1; p=0.120) and age of first delivery and CIN (X^2^= 0.179; df=1; p=0.672) (Table 7).

**Table 1:** Distribution of Districts and VIA and CBE Centers of the Attending Women

Particulars	Number (%)
Name of Districts	
Dhaka	10,614 (65.7)
Narsingdi	764 (4.7)
Narayanganj	566 (3.5)
B.Baria	556 (3.4)
Gazipur	484 (3.0)
Manikganj	431 (2.6)
Comilla	354 (2.2)
Other 57 districts	2,378 (14.7)
Name of VIA and CBE Centres	
Bangabandhu Sheikh Mujib Medical University (BSMMU)	9,733 (60.3)
Upazila Health Complexes (UHCs)	2,832 (17.5)
District Hospitals (DHs)	1,620 (10.0)
Maternal and Child Welfare Centres (MCWCs)	1,072 (6.6)
Medical College Hospitals (MCHs)	489 (3.0)
NGOs	401 (2.5)

*Percentage within parenthesis, ; a) VIA- Visual Inspection of Cervix with Acetic Acid; b) CBE- Clinical Breast Examination

**Table 2:** Demographic and Reproductive Characteristics of Women (n=1,6147)

Characteristics	Categories	Number (%)
Age	Less than 29 years	3,765 (23.3)
	30-39 years	7,187 (44.5)
	40-49 years	3,740 (23.2)
	50 years and above	1,455 (9.0)
Education of Women	No Formal Education	4,223 (26.2)
	Primary (5 years schooling)	7,038 (43.6)
	SSC (10 years schooling)	2,504 (15.5)
	HSC (12 years schooling) and above	1,149 (7.1)
Occupation of Women	House Wife	15,542 (96.3)
	Service holder	567 (3.5)
	Labour	38 (0.2)
Occupation of Husband	Service holder	6,446 (44.1)
	Business	5,411 (33.5)
	Farmer	1,966 (12.2)
	Labour	899 (5.6)
	Driver	728 (4.5)
	Not alive	672 (4.2)
	Unemployed	25 (0.2)
Monthly family income	Upto Taka 3,000	707 (4.4)
	Taka 3,001 - 6,000	2,563 (15.9)
	Taka 6,001 - 10,000	1,858 (11.5)
	Taka 10,000 and above	10,623 (65.8)
	Dependent	396 (2.5)
Parity	Nuli-para	460 (2.8)
	Parity 1-2	8,169 (50.6)
	Parity 3-5	6,746 (41.8)
	Parity 6 and above	772 (4.8)
Age of marriage	Up to 15 years	4,820 (29.9)
	16 to 17 years	7,188 (44.5)
	18 years and above	4,139 (25.6)
Age of delivery	No children	293 (1.8)
	<15 years	1,861 (11.5)
	16-20 years	10,415 (64.5)
	21 years and above	3,578 (22.2)

*, Percentage within parenthesis,; a) SSC- Secondary School Certificate; b) HSC- Higher Secondary Certificate

**Table 3:** Histopathology Results of the Women Who had Colposcopy

Colposcopy Findings	Histopathology Result								Total
	Normal	CIN-I	CIN-II	CIN-III	Carcinoma	Tuberculosis	Report not available	Histopathology not Necessary	
Normal	0 (0.0)	0 (0.0)	0 (0.0)	0 (0.0)	0 (0.0)	0 (0.0)	0 (0.0)	7226 (100.0)	7226 (44.8^a^)
CIN-I	2013 (33.9)	2752 (46.4%)	498 (8.4)	104 (1.8)	7 (0.1)	2 (0.03)	558 (9.4)	0 (0.0)	5934 (36.8^a^)
CIN-II	338 (22.1)	560 (36.7)	369 (24.2)	97 (6.4)	5 (0.3)	0 (0.00)	159 (10.4)	0 (0.0)	1528 (9.5^a^)
CIN- III	20 (10.9)	25 (13.6)	35 (19.0)	69 (37.5)	21 (11.4)	0 (0.00)	14 (7.6)	0 (0.0)	184 (1.1^a^)
Ca-cervix	0 (0.0)	0 (0.0)	0 (0.0)	15 (1.3)	1114 (97.3)	5 (0.4)	11 (0.9)	0 (0.0)	1145 (7.1^a^)
Colposcopically Unsatisfactory	69 (53.1)	14 (20.8)	1 (0.7)	1 (0.8)	2 (1.5)	0 (0.00)	43 (33.1)	0 (0.0)	130 (0.8^a^)
Total	2440 (15.1)	3351 (20.8)	903 (5.6)	286 (1.8)	1149 (7.1)	7 (0.04)	785 (4.9)	7226 (44.8)	16147 (100.0)

*Row percentage within parenthesis

**Table 4:** Sensitivity, Specificity, PPV and NPV of Colposcopy Considering CIN as Diseas

Colposcopy	Histopathology		Total
	Positive	Negative	
Positive	5,671	2,397	8,068
Negative	18	7,316	7,334
Total	5,689	9,713	15,402
Sensitivity	99.7%		
Specificity	75.3%		
PPV	70.3%		
NPV	99.8%		

a) PPV- Positive Predictive Value; b) NPV- Negative Predictive Value;

c) CIN- Cervical intraepithelial neoplasia

**Table 5:** Sensitivity, Specificity, PPV and NPV of Colposcopy Considering CIN2+ as Disease

Colposcopy -	Histopathology		Total
	Positive	Negative	
Positive	1725	954	2,679
Negative	613	12,110	12,723
Total	2338	13,064	15,402
Sensitivity	73.80%		
Specificity	92.70%		
PPV	64.40%		
NPV	95.20%		

a) PPV- Positive Predictive Value; b) NPV- Negative Predictive Value; c) CIN- Cervical intraepithelial neoplasia

**Table 6:** Influence of Demographic and Reproductive Characteristics on Developing Cervical Pre-Cancer and Cancer

Variable	SE	p-value	Exp (B)
Age			
Less than 30 years	0.002	0	1.071
30 years and above			
Education of women			
Illiterate	0.024	0.007	0.938
Literate			
Socio-economic status			
Income < Tk. 10K	0.022	0.014	0.947
Income > Tk. 10K			
Age of marriage			
Less than 18 years	0.01	0.295	0.989
18 years and above			
Age of first delivery			
Less than 18 years	0.007	0.379	0.993
18 years and above			
Parity			
1-3 child	0.017	0.001	1.056
More than 3 Child			

a) SE- Standard error; b) K= 1000

**Table 7:** Assocaition between Demographic and Reproductive Factors with CIN and Non-CIN Population

Variable	Histology Findings		SE	p-value
	CIN	Non-CIN		
Age				
Less than 30 years	1766 (38.9)	4417 (38.1)	0.984	0.321
30 years and above	2774 (61.1)	7190 (61.9)		
Education of women				
Illiterate	987 (21.7)	3236 (27.9)	63.694	0
Literate	3553 (78.3)	8371 (72.1)		
Socio-economic status				
Income < Tk. 10K	1511 (33.3)	4013 (34.6)	2.421	0.12
Income > Tk. 10K	3029 (66.7)	7594 (65.4)		
Age of marriage				
Less than 18 years	3254 (71.7)	8754 (75.4)	24.023	0
18 years and above	1286 (28.3)	2853 (24.6)		
Age of first delivery				
Less than 18 years	1635 (36.9)	4255 (37.3)	0.179	0.672
18 years and above	2797 (63.1)	7167 (62.7)		
Parity				
1-3 Childs	2408 (55.0)	5761 (51.0)	20.338	0
More than 3 child	1973 (45.0)	5545 (49.0)		

*, Column Percentage within Parenthesis, CIN- Cervical intraepithelial neoplasia; K= 1,000, SE- Standard Erro

## Discussion

Development of CIN and its progression to CC is one of the important health concerns worldwide. In a developing country like Bangladesh, the huge load of CC patient is referred to tertiary hospitals and institutions. Treatment of this cancer is expensive and requires radical operative procedures, prolonged hospital stay, and/or radiotherapy/ chemotherapy. This research was carried out in a developing country without facility of nationwide high-quality screening programme and CC vaccination. Therefore, these results are not comparable to the result of a developing country.

Being a large tertiary hospital and major referral centre, BSMMU received a good number of cervical pre-cancer and cancer each year. Trained gynaecologist who have got basic and advanced colposcopy training do the colposcopy of VIA+ve women. Colposcopy findings showed that 10.6 % of the VIA+ve women had high grade disease and 7.1% had CC. However, histopathology report showed lower prevalence of high grade disease (7.4%) and CC prevalence remained unchanged. The sensitivity and specificity of colposcopy were 74.1% and 91.8% respectively. Sensitivity was about three-fourths, so the test is able to detect three-fourths of the people with disease. Among women with colposcopy diagnosed cervical pre-cancer and cancer, the probability of disease was 60.5%. Women who had normal colposcopy findings, the probability of being disease-free were 95.4%.

A significant number of women below 29 years were included in the program and 19.0% of women in this group had high grade disease and 3.7% had CCs. CC screening is a opportunistic screening program in Bangladesh and program does not exclude any married women. Moreover, the legal age of marriage for women in Bangladesh is 18 years, but a large proportion of marriages still take place before the woman reaches her legal age. The mean age at marriage for females is 19.4 in 2011. The BDHS 2014 found that 59 percent of women of 20-24 years were married before age 18. For this, a good number of women below 29 years came for getting the opportunistic screening services and program could not refuse them.

This study depended both on colposcopy and histological findings. There is well known limitations of these procedures as both are nature dependent and may have individual subjective interpretations (Stoler and Schiffman, 2001; Jeronimo and Schiffman, 2006). In developing countries like Bangladesh other tests for improving sensitivity like HPV DNA test or liquid-based cytology (LBC) are expensive and not rational for economic reasons. However, these limitations were minimized by quality assurance by senior experienced Colposcopists. The histopathology reports were also done by competent pathologists who primarily analysed each histopathology slide and then reconciled differences and reported results by agreement.

This study was performed in unscreened population of Bangladesh and found influence of several demographic and reproductive factors on cervical pre-cancer and cancer. Regression analysis showed significant relation of higher age with development of cervical pre-cancer and cancer. It revealed increasing trend of developing high grade pre-cancers in 30 -39 years age group, followed by declined trend to 25.06% during 40-49 years and 10.5% after 50 years of age. On the other hand, there was increasing trend of developing CC, starting from 3.7% at less than 29 years and 43.4% after 50 years of age. As most of the target population remained unscreened, cancer prevalence gradually increased with age reflecting conversion of high grade lesions to cancer among elderly population. With increasing screening this situation is expected to be changed. Östör (1993) also showed a rising trend in unscreened population, as CIN III prevalence peaked in the 55 to 59 age group and declined in the older age groups, and this trend could be due to spontaneous regression of CIN or progression to invasive carcinoma in this age group. In this study, about 80% of high grade pre-cancers and 96% of cancers occurred after 30 years. This finding justifies and supports the government strategy of initiation of screening at the age of 30 years in Bangladesh. About 52.9% of the CC occurs between 30-49 years of age and remaining 43.4% occurred after 50 years of age indicates that both premenopausal and postmenopausal women are equally susceptible to have CC. Only 9% of the VIA positive women were more than 50 years of age and among them about 43.4% had CC. This indicated low participation of elderly women in this opportunistic screening programme and improved participation of this group will improve pickup of more cancer cases. 

A study of Brazil and several studies in India revealed increasing prevalence rate of invasive carcinoma with age (d’Ottaviano-Morelli et al., 2004; Reddy et al., 2013;Ramachandran et al., 2016; Akshatha et al., 2017). A study in Egypt also showed old age as a significant risk factor for CC (El-Moselhy et al., 2016). All these findings indicated that screening should be initiated around 30 years of age and women around 40 years and elderly female should get special emphasis in developing countries during CC screening. 

In the current study, marriages at younger age and 1st childbirth have no independent influence on development of cervical pre-cancer and cancer. However, studies in Iraq and Assam, India reported marriage at or less than 18 years was strongly associated with cervical pre-cancer (Khalaf et al., 2015; Paul et al., 2011) and case control study in Egypt also reported young age at marriage (<18 years) as a significant risk factor for CC (El-Moselhy et al., 2016). 

In the present study, among the demographic and reproductive characteristics, lower level of education (0.007), lower socioeconomic condition (p=0.014), higher parity (p=0.001) had individual influence on development of cervical pre-cancer and cancer. Less than one-fourth of the women had secondary education and above and majority of the women were housewives. This reflected a low level of women empowerment in Bangladesh, which may be related to lack of awareness and less health care seeking behavior making them vulnerable to acquire cervical pre-cancer and cancer. A study in Egypt concluded low education level as an important socio-demographic risk factor for CC (El-Moselhy et al., 2016). Study in Assam reported the influence of economic status, on cervical pre-cancer and cancer (Paul et al., 2011). Policy makers should give special emphasis for improving education and job opportunity of women which should further improve their socio-economic condition and empowerment. 

It was observed that 7518 (56.6%) women having parity more than 3 had significant association with development of cervical pre-cancer and cancer. One large population based longitudinal study in Denmark found parity as an important cofactor for developing high-grade cervical disease (Jensen et al., 2013). A pooled analysis of ten case-control studies by the IARC involving eight developing countries of Africa, South America, Asia, and Europe found that parity increased the risk of both invasive and in-situ carcinoma of the cervix (Munoz et al., 2002). Increased hormone levels and impaired immune response during pregnancy have been suggested as risk factor for cervical pre cancer and cancer (Appleby et al., 2006). Moreover, positioning of the transformation zone on the ecto-cervix for longer period in multiparous women facilitates direct exposure to HPV and potential cofactors (Autier et al., 1996). All these information indicate that parity is consistently associated with CC risk and declining multi-parity should have a positive impact in reducing CC in developing countries like Bangladesh. Bangladesh is a highly populated country and at least 20 million population need screening. The GOB is proactive to provide high quality family planning services for limiting childbirth. However more efforts need to be given on this aspect as parity 3 and above is still common in this country.

Prevalence and risk factors of cervical pre-cancer and cancer in Bangladesh has not been described by any population based study in Bangladesh. This study included information from different districts covering large areas of the country.

Government has taken the stewardship to scale up the screening program all over the country and recently developed CC strategy and costed action plan. This will strengthen the program to serve the women. 

The results from the current should must be considered in light of certain limitations. Firstly, the study was carried out in the colposcopy clinic of BSMMU. All data were kept in prescribed colposcopy register, there were limitation of information about some variables like rural, urban, stage of cancer of the screen positive women. Moreover the information on menopause and tobacco habit were not considered. Secondly, there is a possibility of re-call bias about some data, such as, age of marriage, age of first delivery etc. Furthermore a population based epidemiological study in Bangladesh on CC should be performed to find out the impact of socio-demographic and reproductive factors on CC.

In conclusion, this study found that elderly women and women with low socio-economic condition, low education level and high parity were at higher risk of developing CC. The current national program is opportunistic and continuing at the primary, secondary, and tertiary level health care facilities. In order to improve screening coverage specific health interventions should be carried out. The programme should give special importance to elderly women, women with low socio-economic condition, low education level and high parity during screening to have better detection rate at shorter duration. There are resource limitations to implement CC screening program and special attention to these women will pick up more pre-cancer and early cancer. In addition to this, vaccination program for adolescent girls should be carried out. Moreover, woman’s education and empowerment are important socio-demographic factors and need special attention to improve awareness and health seeking behavior.
